# Osteopontin neutralization increases vitamin D receptors on NKT cells and ameliorates liver fibrosis by promoting their activity

**DOI:** 10.3389/fphar.2024.1484278

**Published:** 2024-11-25

**Authors:** Johnny Amer, Ahmad Salhab, Enas Hussini, Rasha Shweiki, Iman Zahran, Mohammad Far

**Affiliations:** ^1^ Department of Allied and Applied Medical Sciences, Division of anatomy, Biochemistry and Genetics, An-Najah National University, Nablus, Palestine; ^2^ Department of Biomedical Sciences, Faculty of Medicine and Health Sciences, An-Najah National University, Nablus, Palestine

**Keywords:** liver fibrosis, NKT cells, OPN, vitamin D, VDR

## Abstract

**Introduction and Aims:**

Vitamin D has an immunomodulatory property influencing the activity of NKT cells. We aimed to study the impact of osteopontin (OPN), a key driver of fibrosis, on NKT cells’ vitamin D receptor (VDR) and activity alterations.

**Methods:**

Liver fibrosis was induced in BALB/C mice with carbon-tetrachloride (CCl_4_) for 8 weeks with either vitamin D [100 ng/kg] or InVivoMAb anti-mouse OPN [100 μg/kg] 2X/week started at week-4 of CCl_4._ The liver injury profile of serum ALT, AST, and inflammatory cytokines were evaluated. Histopathological findings were assessed via H&E staining and Sirius-Red staining. Fibrotic genes of αSMA, CREBP, and collagen III were assessed using RT-PCR. Fast blood sugar, insulin, liver cholesterol, and triglyceride were evaluated. Liver tissue-resident (tr)-NKT cells were obtained for VDR expressions, molecular pathways of p-STAT1 and P-STAT-5, and activation markers of CD107a and NKp46 using flow cytometry.

**Results:**

Following vitamin D treatment, H&E staining revealed reduced microvascular and macrovascular steatosis, while Sirius-Red staining showed less fibrosis accumulation in liver fibrosis mice than in untreated counterparts. Results were associated with a significant decrease in serum cytokines of IL-β/IL-6/IL-4/OPN/TNF-α and serum AST and ALT by 2-fold and 3-fold, respectively. Fibrotic markers showed an average 1.3-fold decrease in αSMA, CREB, and Col-III in liver fibrosis mice following vitamin D treatment. Quantitated liver cholesterol and triglycerides, serum insulin, and fasting blood sugar ameliorated their levels following vitamin D treatment in liver fibrosis mice. OPN-neutralizing antibody over-expressed VDR on trNKT cells and increased CD107a and NKp46 activities of 3.1 and 3.5 folds, respectively, associated with increasing in p-STAT1 and p-STAT5 phosphorylation. These results were accompanied with a decrease in hepatic-stellate-cell activation markers of αSMA, Col-III, and desmin.

**Conclusion:**

VDR expressions affect trNKT cells activity and could modulate progressions of liver fibrosis. Using an OPN-neutralizing antibody exhibited an antifibrotic effect by alleviating the liver injury profile through NKT cells. It is also suggested as an immunomodulatory target of liver fibrosis.

## Graphical Abstract

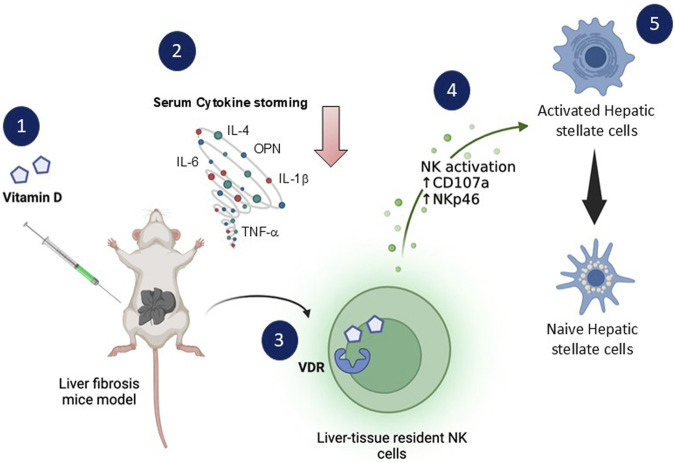

## Introduction

Recently, with the increase in sedentary lifestyles, lack of exercise, obesity, insulin resistance, and others, there has been a dramatic increase in Metabolic dysfunction-associated steatohepatitis (MASH) and chronic liver disease (Younossi et al., 2023). As vitamin D is a crucial prohormone with a well-known impact on calcium homeostasis, it has also come to be understood that it plays a role in cell division and proliferation and has immunomodulatory, anti-inflammatory, and anti-fibrotic effects (Umar et al., 2018). It has recently been suggested that vitamin D receptor (VDR) polymorphism is associated with liver fibrosis chronicity (Triantos et al., 2018). In a previous study, we demonstrated the binary effects of vitamin D on liver fibrosis (Salhab et al., 2020). While vitamin D decreased inflammatory and fibrotic markers in the acute model, it has exacerbated liver fibrosis in chronic condition (Salhab et al., 2020). The Natural Killer (NK) cells in the chronic fibrotic model exhibited reduced expressions of VDR, which was linked to impairment in their activity and inability to kill activated hepatic stellate cells (HSCs), implying that VDR alterations are essential for NK cell activation and, as a result, regulate the advancement of liver fibrosis (Salhab et al., 2020).

Presently, there is a shortage of extensive research concerning the impact of vitamin D on NKT cells (Bychinin et al., 2022; Hutchinson and Pringle, 2022; Yu and Cantorna, 2008) and their associated mechanisms and their potential role in facilitating metabolic profiles (Guo et al., 2020), stimulating immune responses (Martens et al., 2020), or promoting liver injury repair (Luo et al., 2023). Despite the emerging evidence of studies coupling vitamin D to anti-inflammatory and anti-fibrotic effects (Aggeletopoulou et al., 2022), understanding its effect on NKT cells, VDR expression, and the consecutive impact on chronic liver disease progression remains a gap.

Osteopontin (OPN), or SPP1, is a multifunctional glycoprotein that plays a critical role in the pathogenesis of liver fibrosis (Lin et al., 2023). It activates hepatic stellate cells (HSCs), which are critical drivers of fibrosis through their production of extracellular matrix components (George et al., 2022). Elevated OPN levels are often observed in fibrotic liver tissue, contributing to inflammation, tissue remodeling, and the fibrogenic process (Huang et al., 2010). Antagonizing osteopontin has emerged as a potential therapeutic strategy for combating liver fibrosis and cancer (Nardo et al., 2020).

Our research aims to investigate the effects of OPN antagonizing NKT cell activity via vitamin D signaling in the liver fibrosis model.

## Methodology

### Study design and setting

BALB/C male mice model weighted 22–24 ± 0.7 g received care at week 12 of age according to the An-Najah University ethical regulations and NIH guidelines. The institutional animal care ethical committee approved all animal protocols under the ethical number 2024–01–04. The current study includes four different groups of animals mice model and four treatment groups: (1) naïve mice treated with corn oil, which was used as a control (injected i. p twice a week for 8 weeks’ duration), (2) naïve animals treated with pure vitamin D (active form of vitamin D, 1,25(OH)_2_D_3_; Sigma, CAT# 50299-1 MG-F; 100 ng/kg (2.5 ng/mouse) injected i. p twice a week for 4 weeks’ duration, (3) liver fibrosis mice model (treated with CCl_4_ at the concentration of 0.5 μL pure CCl_4_/g body weight for 8 weeks), (4) liver fibrosis mice model treated with pure vitamin D (100 ng/kg) i. p injection twice a week for 4 weeks duration starting from week four of CCl_4_; n = 6 in every group. Potential side effects of vitamin D doses were assessed by detecting calcium serum levels for hypercalcemia using a calcium assay kit, which was colorimetric; ab102505.

In other sets of experiments, four groups of mice were utilized to investigate the role of osteopontin (OPN)-neutralizing antibody in the liver fibrosis mice model. The groups included (1) naive mice treated with PBS, (2) naive mice treated with OPN-neutralizing antibody [InVivoMAb anti-mouse OPN (Cat# BE0373) at a concentration of 100 μg/kg], (3) mice induced with CCl_4_-liver fibrosis and treated with PBS, (4) mice induced with CCl_4_-liver fibrosis and treated with InVivoMAb anti-mouse OPN. OPN-neutralizing antibody was i. p injected twice weekly from week four of CCl_4_ till week eight (n = 6 in every group). Body weights were measured at the start of the experiment and on the sacrifice days in both sets of experiments.

### Histological assessment

The posterior one-third of the liver was dissected and fixed with 4% formalin for 24 h at room temperature and then embedded in paraffin and sliced (7 μm) in an automated tissue processor (microtome). Sections were deparaffinized by xylene immersion. The sections were then rehydrated using a graded series of ethanol concentrations, starting with absolute ethanol and ending with distilled water: Hematoxylin and eosin (H&E) staining evaluated steatosis, necroinflammatory regions, and apoptotic bodies. Sirius red F3B (0.1%) in saturated picric acid (Abcam, Cat# ab150681) was used to visualize connective tissue and collagen depositions. A veterinary pathologist assessed all histopathological findings and reported assessment gradings. For quantification of the area of fibrosis, stained slides were scanned using a Zeiss microscope with image analysis software (ImageJ) to outline the fibrotic regions within the tissue section, and the fibrotic area was calculated by dividing the area of fibrosis by the total area of the fields of view or sections analyzed.

### RNA isolation, cDNA preparation, and real-time PCR

Total cellular RNA (2 μg/μL, purity 98%, determined using a Nanodrop ND-1000 spectrophotometer, Nanodrop Technologies, Wilmington, DE) was isolated from mouse liver tissue samples using 2 mL of TRI reagent (Bio Lab; Cat# 90102331). The samples were centrifuged (14,000 rpm) for 15 min at 4°C, and RNA-containing supernatants were collected. For RNA precipitation, the supernatants were transferred to a new microcentrifuge tube, 0.5 mL of isopropanol (Bio Lab; Cat# 16260521) was added, and the samples were incubated at 25°C for 10 min and centrifuged (12,000 rpm) for 10 min at 4°C. The supernatants were removed, and 1 mL of 75% ethanol was added to the pellets before centrifugation (7,500 rpm) for 5 min. The pellets were air-dried at room temperature for 15 min, 50 μL of DEPC-treated water was added, and the samples were heated for 10 minutes at 55°C.

cDNA was prepared with a High-Capacity cDNA Isolation Kit (R&D; Cat# 1406197). Real-time PCR was performed to quantify αSMA (Rhenium; Cat# Mm00725412), Col III (Rhenium; Cat# Mm00801666), and CREBP (Rhenium; Cat# Mm00446229) gene expression using TaqMan Fast Advanced Master Mix (Applied Biosystems, Cat# 4371130). Gene expression was normalized to the housekeeping genes of GAPDH (Rhenium; Cat# Mm99999915) and NDUFP (Rhenium; Cat# AB-4331182). The cycling conditions for the one-step RT‒PCR involved 40 cycles of 94°C for 30 s, 60°C for 30 s, and 72°C for 1 min, followed by 72°C for 10 min. Data were analyzed using a QuantStudio™ 5 Real-Time PCR System (Applied Biosystems, Cat# A34322), the naïve untreated group is set at 1 and used as a calibrator (Control group) for the experiments and analysis.

Serum and liver biochemical markers and Homeostatic Model Assessment for Insulin Resistance (HOMA-IR) score.

The fasting blood sugar (FBS; Abcam; Cat# ab65333), serum insulin (Abcam; Cat# ab277390), values obtained from mice fasted for 16 h before sacrificing were measured following whole blood samples collection from the heart and centrifuged at 5,000 rpm for 30 min at 4°C. In addition, FBS and serum insulin were used to calculate the HOMA-IR index using the formula: HOMA-IR = fasting insulin concentration (µU/mL) × fasting glucose concentration (mmol/L)/22.5. HOMA-IR values were recorded and calculated for each mouse. Moreover, Serum alanine transaminase (ALT; Abcam; Cat# ab285263), Aspartate aminotransferase (AST; Abcam; Cat# ab263882), liver isolated cholesterol (Abcam; Cat# ab285242), and triglyceride (Abcam; ab65336), were measured using ELISA kits according to the manufacturer’s protocols. All reagents and samples were brought to room temperature (18°C–25°C) before use. One hundred microliters of each standard and sample were added to the appropriate wells and incubated for 2.5 h at room temperature with gentle shaking. The solution was discarded, and the wells were washed four times with X1 wash buffer (300 µL) using a multichannel pipette or auto washer. After each step, the liquid was removed entirely. One hundred microliters of detection antibody were added to each well and incubated for 1 h at room temperature with gentle shaking. One hundred microliters of streptavidin solution were added to each well and incubated for 45 min at room temperature with gentle shaking. One hundred microliters of TMB One-Step Substrate Reagent (Item H) were added to each well and incubated for 30 min at room temperature in the dark with gentle shaking. Finally, 50 µL of Stop Solution (Item I) was added to each well, and the absorbance at 450 nm was immediately read with an ELISA reader (Tecan M100 Plate Reader, An-Najah Central Lab).

### Luminex-magpix tests

A multiplexed sandwich enzyme‐linked immunosorbent assay‐based technology (Cat# MHSTCMAG-70K; R&D Systems) was used to simultaneously determine the concentration of multiple cytokines (IL-1β, IL-4, IL-6, OPN, and TNF-α). Samples from each group of mice were analyzed as instructed by the kit.

### Liver tissue-resident NKT (trNKT) cell isolation

The livers were removed and transferred to Petri dishes containing 10 mL DMEM medium (Biological Industries; Cat# 01–055-1A). Tissues were thoroughly dispersed with a stainless-steel mesh, and the cells were harvested with the medium and transferred to 50 mL tubes containing 10 mL DMEM. The samples were diluted with an equal volume of phosphate-buffered saline (PBS) or cell wash buffer, mixed gently, and layered on top of an equal volume of Ficoll-Paque (GE17-1,440–02; Merck) in a separate tube. The tube was centrifuged at 515 *g* (with gentle acceleration and no braking) for 20 min at room temperature. The middle layer, containing lymphocytes, was collected under sterile conditions and transferred to a new tube. The lymphocytes were washed with PBS or cell wash buffer, gently mixed, and centrifuged at 515 *g* for 10 min to remove residual contaminants. The NKT cells were isolated from the lymphocytes using an NK1.1+ NKT Cell Isolation Kit (Cat# 130–096–513; Miltenyi Biotec).

### Flow cytometry

All used antibodies were incubated with the isolated liver trNKT cells cell suspensions (1:100) at 4°C for 45 min, and cells were later washed 2X PBS with 1%FCS before the secondary antibody (1:100) at 4°C for 45 min if required. Primary antibodies: anti-mouse NCR1 (NKp46) antibody (Abcam; EPR26322-23), anti-mouse LAMP1 (CD107a) antibody (BD Horizon; R718), anti-mouse αSMA (Abcam; ab7817), anti-mouse Col III (Abcam; EPR17673), anti-mouse Desmin (Abcam; ab32362), anti-p-STAT1 (Y701) (ab109457, R&D Systems), and anti-p-STAT5 (S727) (ab32143, Abcam). Secondary antibodies: Goat Anti-Mouse IgG H&L (Cy2^®^) preadsorbed (ab6944) and Goat Anti-Rabbit IgG H&L (Cy3^®^) preadsorbed (ab6939). All stained cells were examined on a flow cytometer (BD LSR Fortessa™, Becton Dickinson, Immunofluorometry Systems) and analyzed by FCS Express 7 by *De Novo* Software for flow cytometry.

### Statistical analysis

Statistical differences were analyzed using a two-way ANOVA, followed by Tukey’s Honestly Significant Difference (HSD) post-hoc test, in GraphPad Prism 9.0 (GraphPad Software, La Jolla, CA). A *p*-value of ≤0.05 was considered statistically significant. The experiment was repeated 3 times, each with 6 samples per group (n = 6), resulting in 18 samples per group across all experiments. For data analysis, we combined the results from these 18 samples within each group to provide a comprehensive overview of the findings. Results are presented as mean ± SD of all 18 mice in the group.

## Results

### Vitamin D ameliorates inflammatory and fibrotic profiles of CCl_4_-induced liver injury mice model

**FIGURE 1:**
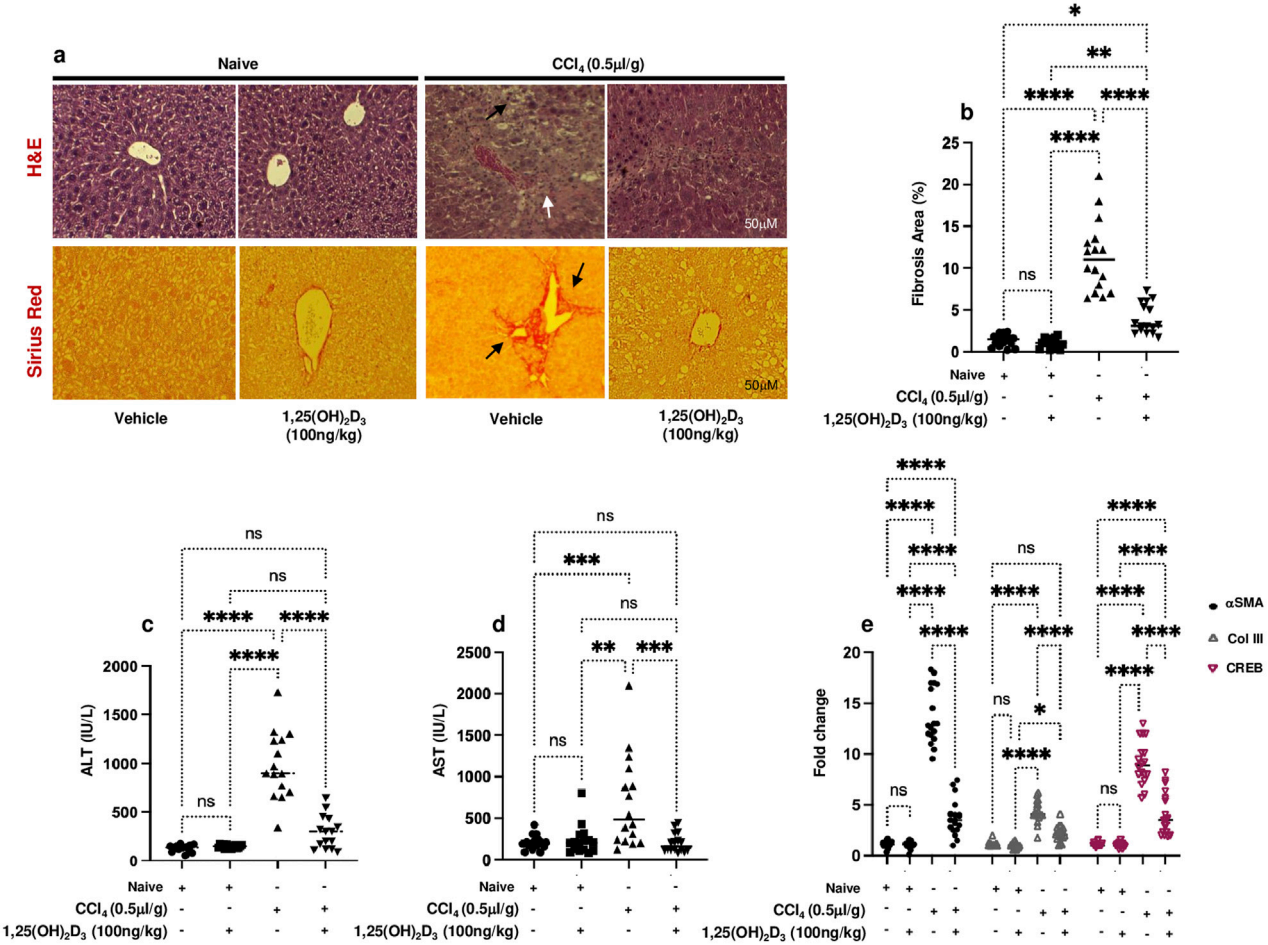
**(A)** Representative images of sections from naïve and liver fibrosis mice treated and untreated with vitamin D stained with H&E (upper row) and Sirius Red (lower row) (original magnification ×20), scale bar = 50 μm. **(B)** Quantitation of the area of fibrosis in liver sections stained with Sirius Red expressed as the percentage of the total tissue area. Quantification was performed using ImageJ software. Serum **(C)** ALT and **(D)** AST levels were determined by ELISA, and liver tissue of **(E)** αSMA, Col-III, and CREB mRNA levels were measured by RT‒PCR. Data are presented as the average ±SD (n = 16 per group in **(B–D)** and n = 18 in **(E)**). Significance was determined using a two-way analysis of variance (ANOVA) with Tukey’s Honestly Significant Difference (HSD) post-hoc test, *p < 0.05, **p < 0.01, ***p < 0.001, ****p < 0.0001.

Given its known anti-inflammatory properties (Umar et al., 2018), understanding the potential therapeutic effects of vitamin D on chronic liver disease is crucial. Our study aimed to investigate how vitamin D supplementation impacts liver pathology in a CCl_4_-induced liver injury mouse model. For this purpose, livers were assessed for liver injury and histopathological findings in the CCl_4_ mice following treatments with vitamin D. Figure 1A shows a representative image of H&E and Sirius Red staining of the liver section obtained from all mice groups. The H&E staining of CCl_4_-treated livers revealed enlarged centrilobular hepatocytes (black arrows) and extensive necrotic areas containing a high number of infiltrating inflammatory cells and steatosis (white arrows), indicating the existence of a persistent CCl_4_ model. The appearance of these histological findings was inhibited in mice treated with vitamin D, and a significant reduction in both microvascular and macrovascular steatosis was observed in the liver fibrosis model. Moreover, Sirius red staining showed increased collagen deposition in perisinusoidal areas in the CCl_4_ mice model (black arrows), and following vitamin D treatment, a decrease in collagen deposition was noted. No significant alteration was observed between naïve and naïve mice treated with vitamin D in both stainings. Figure 1B displays a summary of the averages in the histological scoring system, which was assessed by calculating the fibrosis area percentages. In addition, liver injury enzymes of ALT and AST showed a significant increase in their levels 4-fold in the CCl_4-_treated mice. ALT and AST levels in the CCl_4_ mice receiving vitamin D were reduced by 2-fold and 3-fold, respectively (Figures 1C, D). Moreover, fibrogenic markers of αSMA, collagen III (Col-III), and CREB fibrosis markers assessed using RT-PCR revealed the expected increase in their levels in the CCl_4_-treated mice as compared to their vehicle naïve mice counterparts treated with the oil (*p* < 0.002; Figure 1E). CCl_4_ mice treated with vitamin D demonstrated a significant 1.2 to 1.4-fold decrease in αSMA, Col-III, and CREB liver expressions. Fibrogenic markers were unchanged in the naïve mice treated or untreated with vitamin D (p = ns).

Body weight progression throughout the study showed a 7.2% reduction in mice weights (28 g) following the CCl_4_ administration of their original weight (30 g). In contrast, vitamin D administration inhibited body weight loss and restored their weight, similar to naïve mice. Body weight showed no alteration in naïve groups treated with or without vitamin D (Supplementary Figure S1A). Potential side effects of vitamin D doses were assessed by evaluating serum calcium levels, as hypercalcemia is the most severe side effect of high-dose calcitriol treatment (Umar et al., 2018). Results showed no significant alterations in serum calcium levels, excluding hypercalcemia (Supplementary Figure S1B).

Overall, these findings suggest that vitamin D has promising anti-inflammatory and anti-fibrotic properties that could potentially benefit the management of chronic liver disease.

### Vitamin D improved metabolic assessments and immune-derived proinflammatory cytokines in liver fibrosis mice model

**FIGURE 2:**
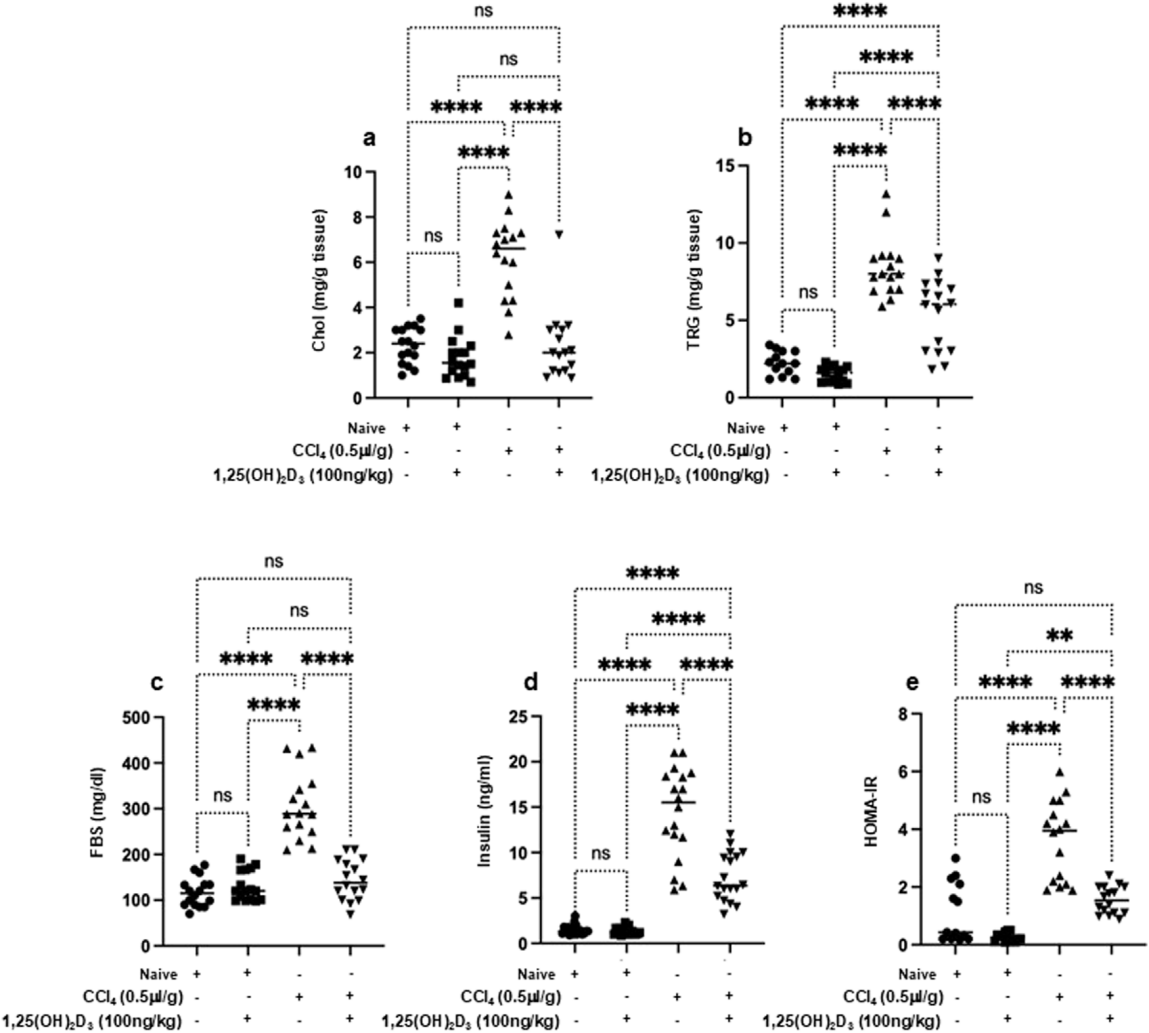
Metabolic markers of the lipid profile of the liver **(A)** cholesterol, **(B)** triglyceride and serum levels of **(C)** fast blood sugar, **(D)** insulin, and **(E)** HOMA-IR were assessed using ELISA following 16 hours of fasting. Data are presented as the average ±SD (n = 16 per group). Significance was determined using a two-way analysis of variance (ANOVA) with Tukey’s Honestly Significant Difference (HSD) post-hoc test, **p < 0.01, ****p < 0.0001.

**FIGURE 3:**
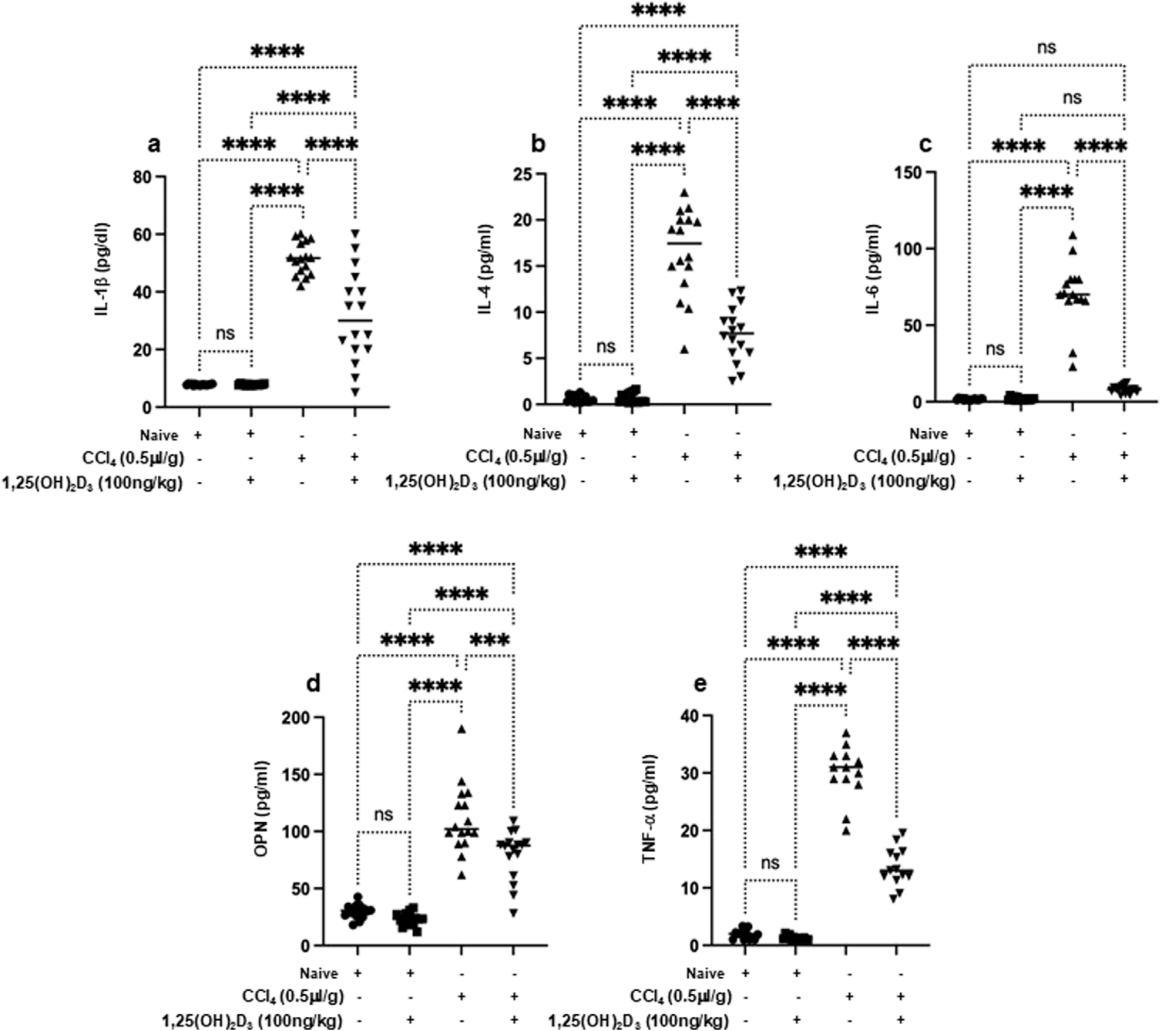
Serum proinflammatory cytokines of **(A)** IL-1β, **(B)** IL-4, **(C)** IL-6, **(D)** OPN, and **(E)** TNF-α were assessed using ELISA following 16 hours of fasting. Data were analyzed using a Quantibody Q-Analyzer and an Excel-based program; results are presented in pg/mL. Data are presented as the average ±SD (n = 16 per group in Figures **(A–C)** and n = 13 in **(D, E)**). Significance was determined using a two-way analysis of variance (ANOVA) with Tukey’s Honestly Significant Difference (HSD) post-hoc test, ***p < 0.001, ****p < 0.0001.

Knockaert L *et al.* showed a significant impact of CCl₄ on liver lipid metabolism and glucose homeostasis and highlighted its potential role in the progression of liver diseases (Knockaert et al., 2012). In our current study, naïve mice exhibited liver cholesterol of 2.1 ± 1.1 mg/g tissue, triglycerides of 2.3 ± 0.87 mg/g tissue, fasting blood sugar of 99 ± 13 mg/dL and serum insulin levels of 2.9 ± 2 ng/ml (Figures 2A–D). CCl₄-treated mice exhibited significantly elevated levels of liver cholesterol of 5.9 ± 1.1 mg/g tissue, triglycerides of 7.3 ± 0.89 mg/g tissue, fasting blood sugar of 299 ± 10 mg/dL and and serum insulin of 16 ± 4 ng/mL (*P* < 0.05); however, with vitamin D treatment, liver cholesterol levels reduced to 1.7 ± 0.4 mg/g tissue, triglycerides to 2.2 ± 0.1 mg/g tissue), fasting blood sugar to 196 ± 7 mg/dL and serum insulin to 6.1 ± 7 ng/ml. These results were statistically significant and indicated the potential effects of vitamin D in improving the metabolic profile in the liver fibrosis model. Naïve mice receiving the vehicle did not exhibit modulation in lipid profile following vitamin D treatments. Moreover, when assessed for HOMA-IR scores, a reduction of 2.1-fold in HOMA-IR score was observed following treatment with vitamin D in the liver fibrosis mice compared to untreated counterparts (Figure 2E). These findings, in part, suggest vitamin D has a potential therapeutic effect in mitigating liver fibrosis association with metabolic disturbances, highlighting its promise as a treatment for impaired lipid and glucose homeostasis conditions. Although the above results conclude the efficacy of vitamin D in alleviating metabolic syndrome associated with liver fibrosis, it still demonstrates involvement of NKT cells in delaying complications of liver fibrosis. Therefore, we sought to assess the effects of vitamin D on the cytokines-derived immune cells. We have previously demonstrated vitamin D’s impact on alleviating liver fibrosis via improving NK cell cytotoxicity and upregulating VDR on NK cells (Salhab et al., 2020). Furthermore, vitamin D has been shown to inhibit monocyte production of proinflammatory cytokines such as IL-1, IL-6, IL-8, IL-12, and TNF-α (Aranow, 2011). However, the effects of vitamin D influencing OPN, a pro-inflammatory cytokine that plays a role in chronic inflammatory and autoimmune diseases (Lin et al., 2023) are not well understood. Moreover, NKT cell-derived OPN was shown as a novel target for treating inflammatory liver diseases (Nardo et al., 2020). Figure 3 shows serum levels of IL-1β, IL-4, IL-6, and TNF-α in addition to OPN. Data showed that naïve mice treated or untreated with vitamin D consistently maintained low serum cytokine levels. In contrast, CCl₄-treated mice exhibited significantly elevated levels of inflammatory cytokines of IL-1β of 4.5-fold, IL-4 of 18-fold, IL-6 of 80-fold, OPN of 2.3-fold, and TNF-α of 7-fold, as compared to naïve mice untreated with CCl_4_ (*p* < 0.05). Remarkably, the liver fibrosis mice treated with vitamin D resulted in a substantial reduction in the levels of the tested cytokines: IL-1β (2.2-fold), IL-4 (1.9-fold), IL-6 (8-fold), OPN (1.45-fold), and TNF-α (1.7-fold) compared to CCl₄ alone. These findings underscore in addition to vitamin D’s potent anti-inflammatory effects, it also reduced OPN levels in liver fibrosis mice, highlighting the importance of vitamin D in modulating immune cells, among them NKT cell recruitment in the inflammatory process.

OPN-neutralizing antibody induced trNKT cells VDR up expressions, and it is crucial for their modulation of liver fibrosis.

**FIGURE 4:**
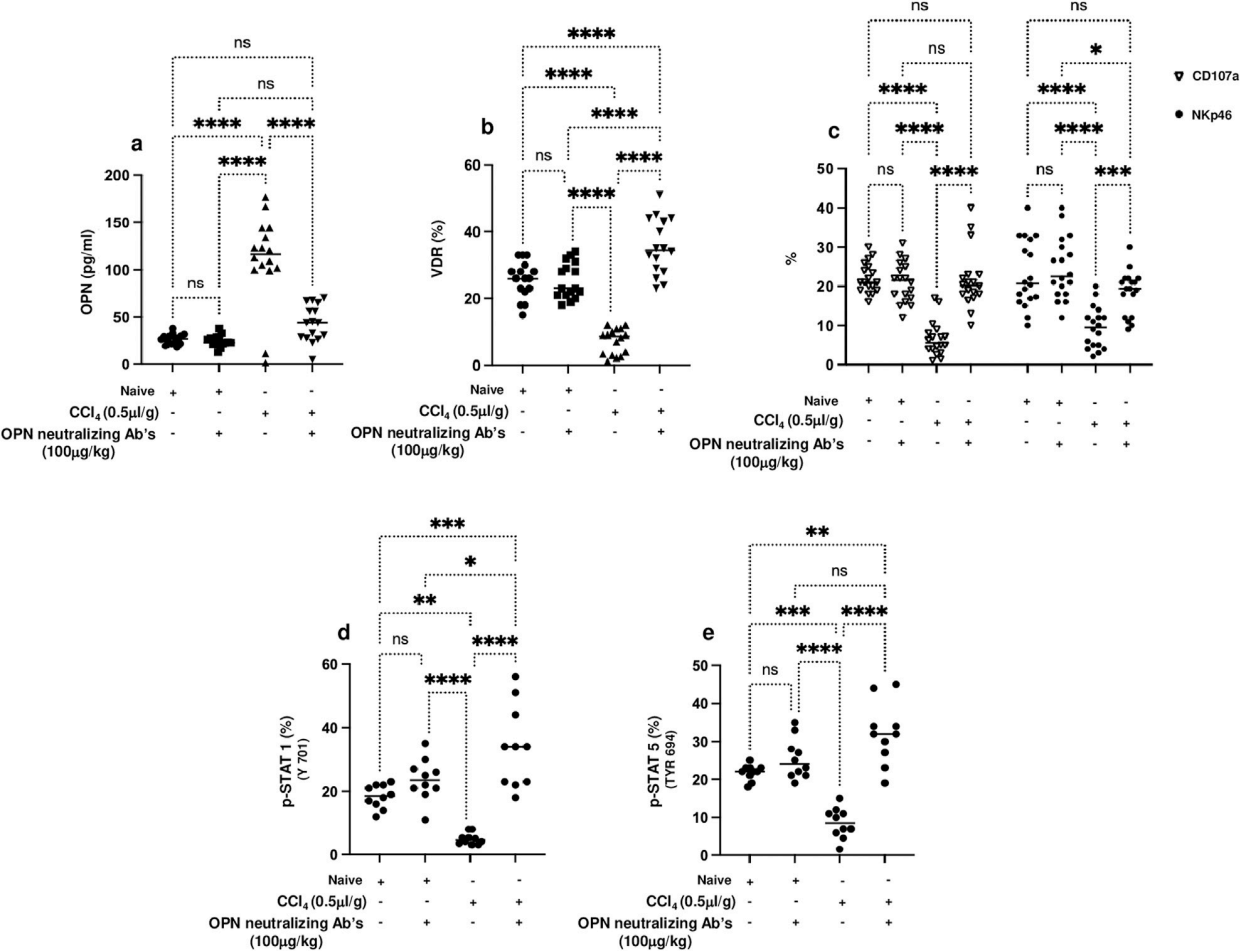
**(A)** Serum levels of OPN were assessed using ELISA following 16 hours of fasting. trNK cells phenotype as NK1.1/CD49a +/CD49b -/CD3+ cells were assessed for percentages of cells expressing **(B)** VDR and **(C)** trNKT activation markers of CD107a and NKp46 and phosphorylated proteins of **(D)** p-STAT1 (Y701), and **(E)** p-STAT5 (Tyr 694) were also detected using flow cytometry. Data are presented as the average ±SD (n = 16 per group in **(A–C)** and n = 10 in **(D, E)**). Significance was determined using a two-way analysis of variance (ANOVA) with Tukey’s Honestly Significant Difference (HSD) post-hoc test, *p < 0.05, **p < 0.01, ***p < 0.001, ****p < 0.0001.

Hence, and as presented in the above results showing elevated OPN in liver fibrosis, we next sought to use neutralizing antibodies against OPN and associate trNKT phenotypic alteration of VDR and activity markers of CD107a and NKp46. Evidence of the effectiveness of the OPN-neutralizing antibody effects was made by assessing serum OPN levels in mice. Figure 4A demonstrates elevated levels of serum OPN in the CCl_4_-induced liver fibrosis mice to levels of a 4-fold increase compared to naïve mice treated or untreated with CCl_4_ (*p* < 0.00001). These levels were reduced significantly in the mice groups treated with OPN-neutralizing antibodies to levels similar to naïve mice (p = ns). To associate these results with trNKT phenotypic alteration, changes in VDR and activity markers were assessed. The isolated liver trNKT cells exhibited a low expression of VDR in liver fibrosis mice, and these expressions were restored to levels comparable to the untreated naïve following the treatment with OPN-neutralizing antibody (Figure 4B). In addition, trNKT cell activity assessed by CD107a and NKp46 was reduced in the liver fibrosis mice model to 4-fold as compared to naïve mice. However, the OPN-neutralizing antibody restored trNKT cell activity expressions to levels comparable to the naïve mice (Figure 4C).

To extend our understanding of the possible molecular mechanism behind the regulation of VDR on NKT cells by OPN, the phosphorylation in STAT1 and STAT5, a well-known protein important in regulating the activity of NKT cells, was assessed following treatment with OPN-neutralizing antibodies. Figures 4D, E show inhibition in STAT1 and STAT5 phosphorylation, respectively, in trNK cells from CCl_4_-induced liver fibrosis mice compared to their Naïve counterparts (*p* < 0.01). Conversely, CCl_4_-induced liver fibrosis mice treated with the OPN-neutralizing antibody showed a 2-fold increase in STAT1 and STAT5 phosphorylation (*p* < 0.00001). STAT1 and STAT5 phosphorylation, VDR, and NKT activation were unchanged in the Naïve mice whether treated or untreated with OPN-neutralizing antibody. These results were associated with amelioration in the liver fibrosis markers of αSMA, Col-III, and Desmin assessed in isolated primary hepatic stellate cells (pHSCs) following treatment with OPN-neutralizing antibody as compared to untreated animals (Supplementary Figure S2, *p* < 0.05). The above data indicate that OPNs are profibrotic cytokines that increase liver fibrosis and are associated with reduced VDR, STAT 1 and STAT5 phosphorylation, and NKT activity. These results also, in part, explain the effects achieved by OPN-neutralizing antibodies only in liver fibrosis and not in naïve mice, indicating its impact on promoting NKT cell activity and ameliorating liver fibrosis.

## Discussion

Several studies showed the effects of vitamin D intervention in attenuating the secretion of inflammatory cytokines induced by liver cirrhosis (Triantos et al., 2018; Salhab et al., 2020; Bychinin et al., 2022). Vitamin D has been shown to inhibit monocyte production of proinflammatory cytokines such as IL-1, IL-6, IL-8, IL-12, and TNF-α (Bui et al., 2021). Still, the impact of vitamin D on NKT cells in liver fibrosis is poorly understood. Moreover, the liver is a significant site of lipid metabolism, and there is a potential link between liver fibrosis and lipid metabolism disorders (Nguyen et al., 2008; Alves-Bezerra and Cohen, 2017). Dysregulation of lipid homeostasis in hepatocytes leads to toxic lipids that result in dysfunctional organelles promoting inflammation, hepatocellular damage, and cell demise (Ekstedt et al., 2015). Our data demonstrated other metabolic markers, such as fasting blood sugar, insulin levels, and the homeostatic model assessment of insulin resistance (HOMA-IR) index, illustrating a significant impairment of glucose homeostasis and insulin resistance in CCl_4_-administered mice. However, vitamin D-treated mice significantly improved in these parameters compared to their untreated groups.

Our current study confirmed the anti-inflammatory effects of vitamin D supplementation on mice with liver fibrosis through reductions in IL-1β, IL-4, IL-6, and TNFα in addition to OPN. Moreover, we directed our study to associate OPN as an additional pro-inflammatory cytokine necessary to recruit T and NKT cells to the liver. Diao et al. also identified NKT cell-derived OPN as a novel target for treating inflammatory liver diseases via augmenting NKT cell activation and triggering neutrophil infiltration and activation (Diao et al., 2004). OPN is known not only as an extracellular matrix protein, supporting adhesion and migration of inflammatory cells, but also as an immunoregulatory cytokin (O'Regan and Berman, 2000). In our study setting, we sought to determine whether vitamin D supplementation could, in part, modulate NKT cells activations and consequently influence liver fibrosis progressions. Vitamin D alleviated NKT cells activity and reduced pro-inflammatory cytokines including OPN levels and these effects were associated with amelioration in liver fibrosis. Results were confirmed through antagonizing OPN and, in addition, induced elevation in VDR on NKT cells. A previous study by Salhab et al. emphasized the importance of VDR for stimulating NK cells and cytotoxic activity in killing activated HSCs and delaying liver fibrosis progressions (Salhab et al., 2020). In our data, NKT cell activity is believed to inhibit Hepatic stellate cells (HSCs) activations and is regulated in part via VDR, and it is associated with increased CD107a and NKp46. NKT cell secretions to cytokines were reduced following treatment with vitamin D, and this is a crucial step in giving them antifibrotic properties, unlike several studies that suggest Many studies suggest that NKT cells promote liver fibrogenesis by producing pro-fibrotic cytokines such as IL-4, IL-13, hedgehog ligands, and OPN; however, NKT cells may also attenuate liver fibrosis under certain conditions by killing pHSCs and by producing IFN-γ (Gao and Radaeva, 2013).

Therefore, it is suggested interventions related to an increase in VDR and vitamin D supplements could increase NKT cells’ expression of VDR in the advanced stages of the disease. After supportive results were shown in the metabolic and fibrotic panel, the experimental findings demonstrated a promising correlation between NKT cells and inflammatory markers of OPN. They highlighted the role of vitamin D in orchestrating these pathways.

## Conclusion

In conclusion, our findings confirm that vitamin D is protective in reducing liver damage and improving liver histology, evidenced by decreased fibrosis and inflammation. These benefits were linked to altered VDR expression on liver trNKT cells; specifically, we observed reduced VDR expression in the untreated fibrotic model and upregulation of VDR in fibrotic mice treated with vitamin D. This suggests a potential connection between increased VDR expression on trNKT cells and the mitigation of fibrosis. If future research confirms the immunomodulatory functions of trNKT cells in this context, they could become valuable targets for novel therapeutic interventions in liver fibrosis. Additionally, using an OPN-neutralizing antibody further mitigates liver injury through the modulation of NKT cells, underscoring its potential as an antifibrotic strategy.

## Data Availability

The datasets presented in this study can be found in online repositories. The names of the repository/repositories and accession number(s) can be found in the article/supplementary material.
